# A case of Aicardi-Goutières syndrome caused by *TREX1* gene mutation

**DOI:** 10.1186/s12884-023-05436-5

**Published:** 2023-02-22

**Authors:** Zheng Chenhan, Shao Jun, Ding Yang, Yin Linliang, Gu Xiaowen, Ji Chunya, Deng Xuedong

**Affiliations:** 1 Center for Medical Ultrasound, The Affiliated Suzhou Hospital of Nanjing Medical University, Suzhou Municipal Hospital, Suzhou, China; 2 Department of Ultrasound, Kunshan Hospital Affiliated to Jiangsu University, Suzhou, China; 3 Center for Reproduction and Genetics, The Affiliated Suzhou Hospital of Nanjing Medical University, Suzhou Municipal Hospital, Suzhou, China; 4 Department of Radiology, The Affiliated Suzhou Hospital of Nanjing Medical University, Suzhou Municipal Hospital, Suzhou, China

**Keywords:** Aicardi-Goutières syndrome, *TREX1*, Microcephaly, Nervous system malformations, Autoimmune diseases of the nervous system, Prenatal diagnosis, Whole-exome genome sequencing

## Abstract

Aicardi-Goutières syndrome (AGS) is a rare genetic disorder involving the central nervous system and autoimmune abnormalities, leading to severe intellectual and physical disability with poor prognosis. AGS has a phenotype similar to intrauterine viral infection, which often leads to delays in genetic counseling. In this study, we report a case with a prenatal diagnosis of AGS. The first fetal ultrasound detected bilateral lateral ventricle cystic structures, and fetal MRI was performed to identify other signs. The right parietal lobe signal showed cerebral white matter abnormalities, and fetal brain development level was lower than that of normal fetuses of the same gestational age. Whole-exome sequencing revealed that the fetus carried the *TREX1*:NM_033629.6:exon2:c.294dup:p. C99Mfs*3 variant, suggesting that the c.294dup mutation of the *TREX1* gene was the pathogenic mutation site, and the final comprehensive diagnosis was AGS1. In this article, we also reviewed the previous literature for possible phenotypes in the fetus and found that microcephaly and intrauterine growth retardation may be the first and most important markers of the intrauterine phenotype of AGS.

## Introduction

Aicardi-Goutières syndrome (AGS) is a rare genetic disorder involving the central nervous system and autoimmune abnormalities, leading to severe intellectual and physical disability with poor prognosis [1]. The main clinical features of AGS are microcephaly, multiple intracranial calcifications, white matter lesions, high levels of interferon-I (IFN-I) in the cerebrospinal fluid (CSF), and frostbite-like skin lesions [2]. However, intrauterine infection indicators are negative. AGS is considered a monogenic disease and can be divided into 7 subtypes according to different pathogenic genes: *TREX1* (AGS1), *RNASEH2B* (AGS2), *RNASEH2C* (AGS3), *RNASEH2A* (AGS4), *SAMHD1* (AGS5), *ADAR1* (AGS6) and *IFIH1* (AGS7). At present, prenatally diagnosed cases of AGS are rare. Here, we report a case diagnosed as AGS1 due to abnormal signs found on prenatal ultrasound combined with imaging, clinical examination and genetic diagnosis.

## Case report

The patient was a 27-year-old pregnant woman, gravida 1 para 0. She and her husband were not consanguineous, and both were usually in good health, with no bad habits such as smoking and drinking and no remarkable family history. Routine maternal serologic screening in the first trimester returned negative results for rubella virus, toxoplasma, human immunodeficiency virus, and hepatitis B and C.

**Fig. 1:**
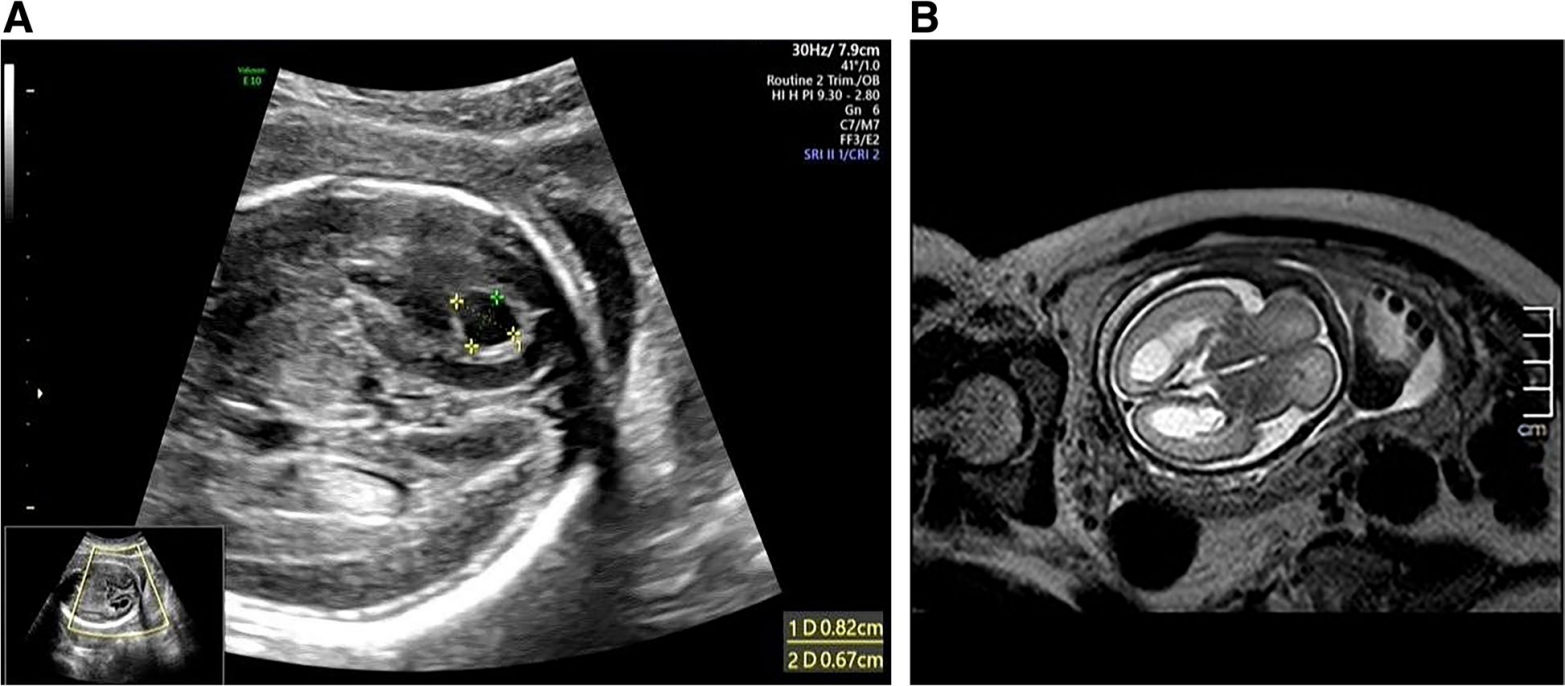
Prenatal ultrasound (**A**) and prenatal fetal MRI (**B**) showed cystic structures in the posterior horn of the fetal lateral ventricle

The pregnant woman underwent a routine mid-trimester fetal ultrasound scan at 24^+1^ gestational weeks. The ultrasound estimated that the fetus’ gestational age was 22w4d in combination with the following fetal growth parameters: fetal biparietal diameter (BPD), 54 mm (< *P*_10_, -1.85 SD); head circumference (HC), 204 mm (< *P*_10_, -1.53 SD); abdominal circumference (AC), 177 mm (< *P*_10_, -1.55 SD); and femur length (FL), 39 mm (*P*_15.4_, -1.02 SD). There were cystic structures at the posterior horn of the lateral ventricles; the structure on the left side was 8.2*6.7 mm (Fig. 1A), and the structure on the right side was 9.1*6.5 mm. A single umbilical artery (SUA) was also detected. Magnetic resonance imaging (MRI) of the fetal brain showed cystic structures around the bilateral lateral ventricles and abnormal signal foci in the white matter of the right parietal lobe. The diameters and sulci of the fetal brain were smaller than those of normal fetuses. Then, the pregnant woman underwent amniocentesis and genetic testing. The results of genetic testing showed a normal chromosome karyotype, and chromosomal microarray analysis (CMA) revealed that there was a heterozygous deletion of 23.9 Mb in the chromosome 5q11.1-q21.3 region of the fetus, which was a variant of uncertain significance (VUS). All indicators of intrauterine infection were negative.

**Fig. 2:**
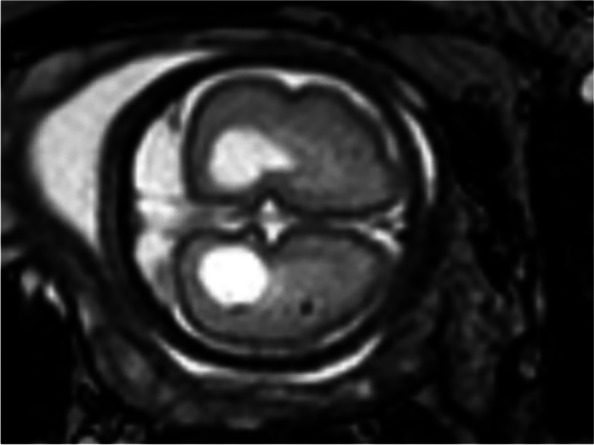
Prenatal fetal MRI showed a short T2 signal in the white matter of the right parietal lobe of the fetus

**Fig. 3:**
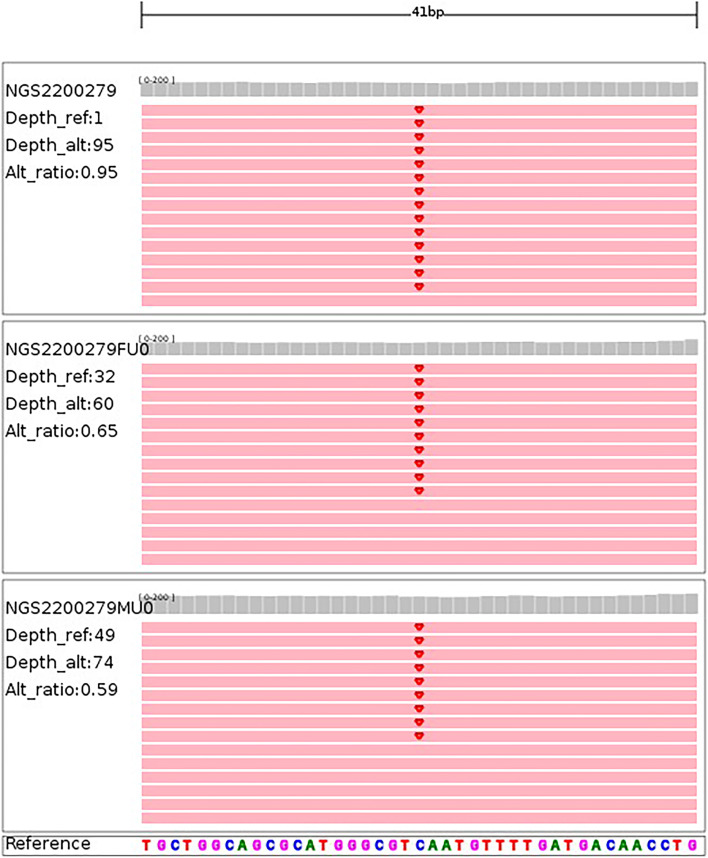
Generation verification result information: *TREX1*:NM_033629.6:exon2:c.294dup:p. C99Mfs*3

The woman continued with the pregnancy and was followed up, the results of which are shown in Table 1. Prenatal ultrasound at 28^+1^ gestational weeks showed that (1) fetal BPD was 63 mm (< *P*_1,_ -2.64 SD), HC was 232 mm (< *P*_1_, -2.99 SD), AC was 205 mm (< *P*_1_, -2.56 SD), and FL was 46 mm (< *P*_3_, -1.94 SD). Combined with fetal growth parameters, the gestational age of the fetus was estimated to be 25^+3^ weeks of gestation. (2) The cystic structures were still present at the posterior horn of each lateral ventricle, and the left and right structures were 12.2*9.3 mm and 11.5*8.6 mm, respectively, which had increased from the previous examination. Fetal MRI showed mild dilation of the bilateral ventricles and cystic structures around them (Fig. 1B), abnormal signal foci in the white matter of the fetal right parietal lobe (Fig. 2), and smaller brain diameter and sulcus development than normal fetuses for more than two weeks. At the same time, according to various fetal growth parameters, the fetal growth trend was slower than that of a normal fetus, manifesting as fetal growth restriction (FGR). In particular, fetal BPD and HC were especially small (less than the 1st percentile) with progressively reduced growth velocity, manifesting as progressive microcephaly. In addition, fetal MRI also provided information about fetal nervous system developmental delay. For further diagnosis, the pregnant woman chose whole-exome genome sequencing, and the results showed that the fetus carried the *TREX1*:NM_033629.6:exon2:c.294dup:p. C99Mfs*3 homozygous variant. The mutation occurred in the second exon of the NM_033629.6 transcription of the *TREX1* gene, resulting in a frameshift mutation (cysteine mutated to methionine) at the 99th amino acid of the coding protein. After a frameshift mutation of 2 amino acids, translation was terminated, and the number of missing amino acids was greater than 10%. Single-nucleotide variant (SNV) and insertion‒deletion (InDel) results showed that the subject had a homozygous mutation of c.294dup in the *TREX1* gene, and both father and mother had heterozygous mutations. Therefore, the mutation originated from the parents (Fig. 3). According to the American College of Medical Genetics and Genomics rating guidelines, these variations were pathogenic. The final comprehensive diagnosis was Aicardi-Goutières syndrome type 1 according to the combined clinical, imaging, and genetic findings. Considering the poor prognosis of individuals with Aicardi-Goutières syndrome type 1, the pregnancy was terminated at 33 weeks of gestation after evaluation by multidisciplinary consultation. The study obtained the informed consent of both spouses, and the informed consent form was signed for all checks and treatments.

**Table 1:** Fetal growth parameters during follow-up

Fetal growth parameters during follow-up										
	BPD/mm	Percentile/SD	HC/mm	Percentile/SD	AC/mm	Percentile/SD	FL/mm	Percentile/SD	EFW/g	Percentile/SD
24w1d	54	3.2/-1.85	204	6.3/-1.53	177	6.1/-1.55	39	15.4/-1.02	514	0.5/-2.58
27w4d	62	0.6/-2.53	230	0.4/-2.69	205	1.6/-2.15	45	2.8/-1.91	759	0/-3.72
28w1d	63	0.6/-2.53	232	0.1/-2.99	205	0.5/-2.56	46	2.6/-1.94	779	0/-4.10
30w1d	65	0/-3.59	246	0/-3.40	230	2.2/-2.01	53	14.2/-1.07	1067	0/-3.29

## Discussion

AGS was first discovered and described by Jean Aicardi and Francoise Goutières in 1984 [3]. Genetic testing is an essential method for the diagnosis of AGS. AGS is genetically heterogeneous. With the advanced development of genetic testing technology, 7 related pathogenic genes have been revealed thus far, including *TREX1* (22%), *RNASEH2B* (36%), *RNASEH2C* (12%), *RNASEH2A* (5%), *SAMHD1* (13%), *ADAR1* (6%), and *IFIH1* (3%), corresponding to AGS types 1–7, respectively [4]. Most genes show a primarily autosomal recessive inheritance pattern, but *IFIH1*, some types of *ADAR* and *TREX1* have an autosomal dominant inheritance pattern [5]. In a multicenter study, Uggenti et al. found that mutations in *LSM11* and *RNU7-1* can also lead to AGS and named them AGS type 8 and type 9 [6].

### Prenatal manifestations and previous literature reports

Due to the poor prognosis of AGS and the lack of effective treatment measures, we reviewed the previous literature to determine whether there is a specific fetal phenotype. Using the key word ‘Aicardi Goutières syndrome’, a comprehensive search was performed in PubMed, Embase, and Web of Science for references to prenatal characteristics of AGS up to August 1, 2022, without regional or language restrictions. Finally, a total of 15 papers were enrolled, including 8 studies in which abnormalities were found prenatally [4, 7–13] and 7 studies in which microcephaly or intrauterine growth retardation was found at birth [2, 14–19]. Ten cases were detected prenatally, including 6 cases with microcephaly [1–3, 7, 8], 2 cases with FGR [7, 8], 5 cases with intracranial calcification [4, 7, 8, 10–12], 3 cases with edema and ascites [4, 7, 11], 2 cases with extraventricular cystic structure [8, 11], 2 cases with ventriculomegaly [8, 12], and 1 case with ventricular asymmetry[12]. A total of 24 cases were found to be abnormal at birth, including 12 cases of microcephaly [2, 14, 15, 18, 19] and 21 cases of FGR [2, 15–17, 19].

In this case, the proband had a *TREX1* mutation, and the clinical phenotype of FGR, progressive microcephaly, and central nervous system developmental delay occurred in the second trimester, especially progressive microcephaly (HC from < *P*_10_ to < *P*_1_), which may be associated with delayed neuronal development. In the literature review, among 10 fetuses that were diagnosed prenatally, only 2 cases showed FGR. Interestingly, among 24 infants diagnosed after birth, 21 cases were found to have a birth weight below the 10th percentile, and 12 cases had microcephaly. Combined with clinically similar symptoms that often appear in children with AGS, we believe that some AGS cases may have the above manifestations in the third trimester; however, there is a large gap between the number of reviewed cases and the number of currently reported AGS cases internationally, and a large sample study is needed to verify this hypothesis. In addition, if there are no other abnormalities in the second or third trimester, doctors usually do not pay excessive attention to developmental delay and microcephaly, which results in delayed diagnosis. Moreover, in addition to AGS, FGR and microcephaly are often the earliest manifestations of some chromosomal abnormalities [20–25]. FGR can be found in triploids, 22q11.2 microduplication syndrome, and other abnormalities, while microcephaly can be found in triploids, Smith‐Lemli‐Opitz syndrome, microcephaly with pontine and cerebellar hypoplasia (MICPCH), and others. Triploidy is often combined with abnormalities in other systems and can be found prenatally. However, many genetic syndromes often show some soft index abnormalities only during pregnancy (for example: one or more growth parameters are lower than normal fetuses at the same gestational age), but they may lead to poor prognosis, so we emphasize the importance of strengthening follow-up for early detection of such genetic syndromes.

### Imaging performance

AGS has the following characteristic imaging features: (1) Intracranial calcification is the main feature of the disease and is seen in most patients with AGS. (2) Abnormal white matter is shown as hyperintensity on T2-weighted imaging; white matter involvement is most commonly diffuse or predominantly frontotemporal, but periventricular or patchy involvement can also occur. (3) Brain atrophy includes ventriculomegaly and sulci enlargement [2, 26]. In this case, fetal MRI showed an abnormal signal focus with low T1 and T2 signal in the white matter of the right parietal lobe of the fetus near the lateral ventricle. Combined with the common features of the syndrome, the possibility of calcification was finally considered. Acute hemorrhage can also show the same appearance on MRI [27], but it changes over time. In our case, the foci showed the same appearance at follow-up during the pregnancy. Fetal MRI is limited in prenatal application due to the long examination time and susceptibility to fetal movement. Prenatal ultrasound still plays a dominant role in prenatal examinations. We believe that in some cases with AGS, intracranial calcification exists in utero; however, it is a challenge for prenatal ultrasound to identify it, which may be because in the early stage of disease, the calcification is too small to be detected by ultrasound. Moreover, the near field of the sound beam limits the display rate. In our literature review, only 5 cases were described as having intracranial calcifications. Two of them [4, 7] were found at autopsy after induction of labor, and 3 cases did not specify the method of detecting calcification.

In this study, the most prominent manifestation of the fetal central nervous system was the cystic structure in the posterior horn of the lateral ventricle, which may be considered a subependymal cyst [28]. Currently, fetal subependymal cysts are mainly considered to be caused by intrauterine infection. However, the intrauterine infection indicators of the pregnant woman in this study were negative. As mentioned above, AGS mostly has an intrauterine viral infection-like phenotype, and the pathophysiological mechanism of the central nervous system may overlap, so it is considered a subependymal cyst. Unfortunately, no pathological autopsy was performed on the infant after induced labor, and therefore, there is no pathological evidence for the nature of this cystic structure. In previous reports of AGS [8, 11], fetuses with periventricular cystic structures similar to those in this study were also described, but specific diagnosis was also absent.

### Genetic analysis

In this study, whole-exome sequencing (WES) technology was used, and the *TREX1*:NM_033629.6:exon2:c.294dup:p. C99Mfs*3 variant was detected in the sample. The variant is pathogenic because it causes a reading frame shift and may result in premature termination of the protein-coding sequence. In previous reports of AGS [3, 29], likely pathogenic variants were identified at the trans position of this variant. In this study, WES provided more genetic information than karyotyping and chromosomal microarray analysis (CMA). In recent years, with the rapid development of WES, large-scale prospective studies have suggested that in fetuses with structural abnormalities, WES can detect more pathogenic-related gene variants. In cases with negative karyotyping and CMA results, WES can improve the detection rate by 8.5%-10%; provide accurate diagnosis, treatment plan and prognosis information; and promote prenatal and postnatal care [30, 31].

The *TREX1* gene is located at chromosome 3p21 and encodes three-prime repair exonuclease 1, which is an essential exonuclease in mammalian cells, and numerous in vivo and in vitro data have shown its participation in immune regulation and genotoxicity remediation [32]. Mutations in *TREX1* lead to protein inactivation, resulting in excess interferon-1, leading to autoimmune disease. High levels of interferon-I may harm the nervous system, resulting in symptoms of AGS; therefore, AGS is also considered to be an autoimmune disease with abnormal interferon-I levels [33, 34]. *TREX1* variants are associated with autoimmune and inflammatory diseases, including AGS, familial chilblain lupus, systemic lupus erythematosus, and retinal vasculopathy with cerebral leukodystrophy. Unlike AGS, the symptoms of several other diseases are relatively mild with a later onset time [35]. In this case, the proband had a series of abnormal manifestations during the fetal period, consistent with the phenotypic characteristics of AGS. *TREX1*-related AGS gene mutations are mostly inherited through an autosomal recessive pattern, children usually herald a complete loss of protein function, and heterozygous parents of patients in such families are usually healthy, as was the case in this study.

In conclusion, we present a detailed case of AGS caused by a TREX1 mutation, enriching the genetic database and prenatal phenotype. By reviewing the literature, we found that microcephaly and intrauterine growth retardation may be the most important phenotypes for intrauterine onset of AGS. Microcephaly and FGR are also phenotypes of some rare syndromes, we also emphasize the importance of follow-up of fetal growth parameters for earlier detection of genetic syndromes, and suggest WES to provide a more accurate diagnosis.

## Data Availability

All data generated or analysed during this study are included in this published article. All authors have unanimously agreed to make publicly available all images and inspection reports included in this article, available from this article.
